# Factors Affecting Length of Stay of Patients in a General Psychiatric Hospital in Bahrain: A Retrospective Study

**DOI:** 10.7759/cureus.65525

**Published:** 2024-07-27

**Authors:** Mazen K Ali, Fatema Hasan, Fatima M Shaheen

**Affiliations:** 1 Psychiatry, Psychiatric Hospital Bahrain, Manama, BHR; 2 Psychiatry, Arabian Gulf University, Manama, BHR

**Keywords:** bahrain, psychiatric hospital, psychiatry, psychiatric disorders, length of stay

## Abstract

Background: Length of stay (LOS) in a psychiatric facility can be used as a measurement of the quality of healthcare. Prolonged stays impact the quality of life of psychiatric patients as well as have a huge burden on healthcare expenditures.

Materials and methods: A retrospective study targeting 153 patients admitted to a general adult ward in a psychiatric hospital in Bahrain, with the final diagnosis based on ICD-10 criteria. The collected data was analyzed using IBM SPSS Statistics for Windows, Version 26 (IBM Corp., Armonk, NY).

Results: The median LOS was 22 days. LOS among schizophrenia and schizoaffective disorder as well as bipolar affective disorder was significantly longer than other groups. There was no significant difference among groups in terms of gender, age, marital status, social class, and alcohol or substance abuse. The presence of extrapyramidal side effects, history of electroconvulsive therapy (ECT) and the use of restraints during admission were associated with longer LOS. A higher number of previous admissions and number of current medications given during admission in the psychiatric hospital predicted a longer stay in the hospital.

Conclusion: Future studies should focus on the effect of better treatment options as well as occupational rehabilitation in ensuring better outcomes for inpatients as well as shorter stays in a psychiatric hospital.

## Introduction

Mental disorders are a major cause of disability and a global burden. The worldwide prevalence of mental illness is predicted to increase [1]. The severity of mental disorders varies immensely and is managed accordingly. Worldwide, patients who often require admission to a psychiatric facility predominantly have severe mental disorders like schizophrenia, bipolar affective disorder as well as major depression [2]. Length of stay (LOS) in a psychiatric facility can be used as a measurement of the quality of healthcare [3]. Prolonged stays impact the quality of life of psychiatric patients as well as have a huge burden on healthcare expenditures [4]. LOS is defined as the number of days in one episode of hospitalization starting from the day of admission and ending when the patient is discharged from the ward [5].

There is only one public psychiatric hospital in Bahrain with approximately 296 beds and an annual average of 1,195 inpatient admissions [6]. A study showed that Bahrain has approximately 30 psychiatric beds per 100,000 individuals, one of the highest proportions among Arab countries as well as a substantial increase in the number of psychiatrists, psychiatry nurses, psychologists, and social workers [7].

Studies investigating factors affecting the length of stay in a psychiatric hospital yielded variable results across different regions worldwide. Studies done in the US showed that mood and psychotic disorders, female gender, and history of electroconvulsive therapy (ECT) contributed to a longer hospital stay [8]. In addition, a study in the UK showed that lengthier hospital stays with a median of 19 days are heavily influenced by male gender, being diagnosed with psychotic disorder, ethnicity, and unemployment [5]. Another study done in the Emirates with an average LOS of 14.52 days showed that substance use disorders, psychotic disorders, and older age were associated with longer stays in the hospital [9]. This is the first study in Bahrain to identify sociodemographic and clinical characteristic factors that affect LOS in a general psychiatric hospital in Bahrain. This will improve our future approach to treating psychiatric patients to ensure shorter stays in a hospital as well as provide psychiatric care more cost-effectively.

## Materials and methods

A retrospective study included 153 patients admitted in general adult wards in Psychiatric Hospital, Ministry of Health, Bahrain between January 2022 and January 2023. Patients were admitted through the emergency department or psychiatry outpatient clinics. The three primary managements offered for patients were pharmaceutical treatment, psychoeducation, and psychotherapy.

The sample size was determined with a 95% confidence level (alpha error of 0.05) and accuracy of 0.08 using the following formula:

$N = \frac{z^{2}*P(1-P)}{d^{2}}$

The exclusion criteria were the cases of ages above 65 and younger than 18.

Data were extracted from patients' electronic files and documented into a Microsoft Excel 16 (Microsoft Corporation, Redmond, WA), spreadsheet then coded and entered into statistical software. Recorded data included demographic data such as age, gender, marital status, occupation, and alcohol and substance abuse, along with information related to hospitalization, including discharge diagnosis, history of use of restraints, presence of extrapyramidal side effects, patient condition at discharge, number of medications given, added to that, history of suicidal attempts, number of previous admissions, history of ECT, and number of comorbid medical and psychiatric conditions. The cases were diagnosed according to ICD-10 criteria by a consultant psychiatrist [10].

The collected data was analyzed using IBM SPSS Statistics for Windows, Version 26 (IBM Corp., Armonk, NY). Descriptive statistics such as measures of frequencies and percentages were computed and presented. Nonparametric tests were used to analyze significant effects on LOS such as the Mann-Whitney U test, the Kruskal-Wallis test, and Spearman's correlation. P-value <0.05 was considered to be statistically significant. 

Verbal consent was obtained from participants before retrieving any sociodemographic or clinical data from their health records. Ethical approval was taken from the Research Committee for Government Hospitals, Ministry of Health, Bahrain to conduct this study (approval no.: 24-220224, dated February 22, 2024).

## Results

One hundred and fifty-three patients were included in our study; 63 (41.2%) were 18-30 years old, the majority were males (93; 60.7%) and most of them followed the Muslim faith (132; 86.3%) (Table 1). Sixty-six (43.1%) were unemployed. Only 44 (29.1%) were non-Bahraini.

**Table 1:** Sociodemographic characteristics. SC: Social class.

Sociodemographic characteristics	Number of patients	%
Gender		
Female	60	39.2
Male	93	60.7
Marital status		
Married	59	38.6
Single	79	51.6
Divorced	12	7.8
Widow	1	0.7
Missing	2	1.3
Religion		
Muslim	132	86.3
Christian	12	7.8
Hindu	6	3.9
Missing	3	2
Employment		
Employed	62	40.5
Unemployed	66	43.1
Retired	18	11.8
Student	7	4.6
Nationality		
Bahraini	109	71.2
Non-Bahraini	44	28.8
Social class		
SC1	0	0
SC2	18	12.1
SC3	41	27.5
SC4	40	26.8
SC5	39	26.2
Missing	11	7.4
Age		
18-30	63	41.2
31-40	42	27.5
41-50	23	15.1
51-60	21	13.8
61-65	4	2.6

Regarding their marital status, 59 (38.6%) were married and 79 (51.6%) were single. According to the Hollingshead and Redlich scale (Table 2) [11], most of the patients were of social class 3 (27.5%) and social class 4 (26.8%).

**Table 2:** Cultural characteristics and class status (Hollingshead and Redlich scale) Source: [11]

Social class (SC)	
SC 1	Community business and professional leaders.
SC 2	Education beyond high school, occupation as manager-lesser ranking professional.
SC 3	High school graduate, administrative and clerical job.
SC 4	Education less than high school and more than primary level working class, semi-skilled, and skilled.
SC 5	Education primary school and less, unskilled workers, or unemployed.

Most of the cases were diagnosed with psychotic disorders including schizophrenia 54 (35.2%), acute psychotic episodes (30; 19.6%), schizoaffective disorder (11; 7.2%), delusional disorder (3; 2%) and drug-induced psychosis (3; 2%). On the other hand, 35 (22.9%) patients suffered from bipolar affective disorder. Patients with a diagnosis of depression were only eleven (7.2%). See Table 3.

**Table 3:** Participant's diagnosis.

Diagnosis	Number of patients	%
No mental disorder	1	0.7
Depression	11	7.2
Bipolar affective disorder	35	22.9
Schizophrenia	54	35.2
Schizoaffective	11	7.2
Delusional disorder	3	2
Acute psychotic episode	30	19.6
Drug-induced psychosis	3	2
others	5	3.3

The median LOS was 22 days. LOS among schizophrenia and schizoaffective disorder, as well as bipolar affective disorder, was significantly longer than other groups (p-value = 0.000). However, we found no significant difference among groups in terms of gender, age, marital status, social class, and alcohol or substance abuse. The presence of extrapyramidal side effects, a history of ECT, and the use of restraints during admission were associated with longer LOS (p-values 0.029, 0.000, and 0.015 respectively). Patients who improved stayed longer than others who went away without leave or signed against medical advice (p-value = 0.008) (Table 4).

**Table 4:** Relationship between median length of stay (IQR) and patient-related variables. * p-value <0.05 is considered statistically significant. EPS: Extrapyramidal symptoms, ECT: Electroconvulsive therapy, SC: Social class, AWL: Away without leave, DAMA: Discharge against medical advice, IQR: Interquartile range.

Characteristics	Median length of stay (IQR)	p-value
Gender	Male 24 (15-35.5)	0.527
	Female 20 (10-42)	
Age	18-30 20 (13-35)	0.321
	31-40 22 (11.5-34.5)	
	41-50 20 (13-30)	
	51-60 30 (19.5-60)	
	61-65 43.5 (18-60)	
Nationality	Bahraini 28 (17-46.5)	0.000*
	Non-Bahraini 14(8-20)	
Marital status	Married 20 (8-30) Single 27(15-39)	0.119
	Divorced 27.5 (14.7-56.2)	
	Widow 30 (30-30)	
Employment	Employed 16.5 (8-22)	0.000*
	Unemployed 30 (20-49.75)	
	Retired 27.5 (14.5-60)	
	Student 19 (12-21)	
Social class	SC2 24.5 (14-35.5)	0.151
	SC3 28 (15-35)	
	SC4 21.5 (13.75-46.75)	
	SC5 17 (8-30)	
Religion	Muslim 27 (15-42) Christian 9 (6.2-14) Hindu 16.5 (11.7-19.2)	0.000*
Discharge diagnosis	Schizophrenia 30 (20.2-42.2)	0.000*
	Bipolar affective disorder 28 (17-45)	
	Depression 20 (7-37)	
	Schizoaffective disorder 38 (20-120)	
	Acute psychotic episode 14 (7-19)	
	Delusional disorder 17	
	Drug-induced psychosis 17	
	Others 14 (12-17.5)	
Smoking	Yes 28 (20-54)	0.031*
	No 20 (13-32)	
Alcohol	Yes 25 (13-42)	0.914
	No 21.5 (13.7-35.5)	
Substance	Yes 22 (13.5-41.5)	0.665
	No 21 (13.37.7)	
Extrapyramidal side effects (EPS)	Yes 34 (16.5-66)	0.029*
	No 21 (13-32.5)	
Outcome	Improved 24 (15-41)	0.008*
	AWL 23 (10-36.7)	
	DAMA 7 (2.5-20.5)	
	Referred 14	
History of suicide	Yes 31 (13.5 -51)	0.165
	No 21 (13-33)	
Electroconvulsive therapy (ECT)	Yes 60 (43.5 -135)	0.000*
	No 20 (13-33)	
Restraints	Yes 30 (17-57)	0.015*
	No 20 (12.2-32.7)	

According to the results of Spearman's correlation test, a higher number of previous admissions and a number of current medications given during admission to the psychiatric hospital predicted a longer stay in the hospital (Table 5).

**Table 5:** Relationship between the quantitative variable and length of stay. * p-value <0.05 is considered statistically significant.

Characteristics	Spearman's correlation coefficient	p-value
Comorbid psychiatric disorders	0.079	0.341
Comorbied medical conditions.	0.176	0.031*
Number of previous admissions	0.405	0.000*
Number of medication	0.369	0.000*

## Discussion

According to the results, factors that influenced the LOS are diagnosis, signing against medical advice, number of previous admissions, medication side effects particularly extrapyramidal symptoms, number of medications, and ECT. These factors are analyzed in this discussion.

Achieving optimal quality of care in addition to using medical resources efficiently through optimizing the LOS in a psychiatric hospital has been an ongoing topic of research in healthcare [12]. International recommendations advocate for a shorter hospital stay as discharge should be made at the point of stabilization [13]. Therefore, studies show that in stable patients, discharge to community care is beneficial compared to long-stay admission, therefore, continuation of management in a less restrictive pattern is recommended [14]. According to the statistical analysis of our study, patient diagnosis contributed significantly to LOS. Patients who were diagnosed with schizoaffective disorder, schizophrenia, and bipolar affective disorder had a longer LOS with median days of 49, 30, and 29 respectively. This finding backs up existing literature, where similar results were found in a study done in Ethiopia [15]. It showed that patients with psychotic disorders and bipolar affective disorders had the longest hospital stay with a median of 22 days. The complex nature of psychotic disorders as well as bipolar affective disorders prevents quick recovery, which can explain our results. This is significant in our region because a study done in Bahrain showed that most patients admitted to in-patient wards suffer from psychotic disorders (66.28%) [16].

LOS was also influenced by the patients and their families’ understanding of the nature of their disease as well as the treatment course in the hospital. Patients who were discharged against medical advice (DAMA) had the lowest average LOS of seven days compared to the median of 22 days for patients who received full treatment until remission from their illness. This has been an escalating issue of concern, as incomplete remission with shorter LOS can exponentially increase the risk of relapse, re-admission, and loss of treatment efficacy in psychiatric patients [17,18]. Exploring reasons behind the increased rate of DAMA in psychiatry hospitals in comparison to other specialties has been an expanding area of research. Research conducted in an Iranian psychiatric facility reported the average LOS for patients who signed against medical advice was 9.5 days. Reasons behind DAMA can be attributed to factors related to dissatisfaction with the hospital environment, inadequate healthcare providers' care, and patients’ sense of improvement [19].

A fundamental variable taken into consideration when investigating LOS is the number of previous admissions. In our study, it contributed significantly, where results showed that an increased number of previous admissions correlated to lengthier stays. This result has been consistent with a systematic review study done in the United States [20]. It suggests that the current LOS can be influenced by previous admissions as recent as two years. The number and length of previous admissions can also influence the future frequency and intensity of relapses indicating a more severe illness.

In our study, patients who experienced medication-induced extrapyramidal symptoms had considerably lengthier hospital stays. Other studies show that the presence of side effects can hinder the patient’s acceptance and response to treatment [21]. The presence of extrapyramidal side effects can lengthen a patient’s stay by requiring further observation and treatment in the ward. This might help guide our future use of medications, especially antipsychotics with fewer side effects. Other factors that may be indicative of illness severity including history of ECT, number of medications prescribed during admission as well as the use of restraints during admission were statistically significant. Polypharmacy and history of ECT are reflective of treatment resistance. Comparable results in a recent Czech study with an average LOS of 1.36 years have shown that patients who are treatment-resistant with at least two or more antipsychotics from different classes are a fundamental predictor of longer stay [22].

In contrast to previous findings, there was a lack of association between gender, age, and marital status with the LOS in a hospital. Replicable findings have been found in a Brazilian study [23]. On the contrary, female gender and older age were found to be influential on the LOS in other studies. The insignificance of age in our study can be attributed to the fact that patients who were older than 65 were excluded from our study.

We believe that our results can be used to decrease the LOS in a psychiatric hospital for patients in future admissions. This can be done by contributing more time to educate patients and their families about the diagnosis and medications. Our results show that patients diagnosed with schizophrenia and psychotic disorders experience longer hospital stays, this can be shortened by introducing more patients to long-acting anti-psychotic medications to prevent relapse. Using clozapine for treatment-resistant patients can help shorten their stay in the hospital. In addition to that, introducing more patients to second-generation antipsychotics can help reduce extrapyramidal side effects during in-patient admissions.

Limitations

The study includes multiple limitations. It is a retrospective design which means that illness severity at the time of admission could not be assessed. In addition, the present study did not include patients less than 18 years old or older than 65 years old; exclusion of these age groups affects the significance of the study results. Moreover, data was retrieved from one psychiatric hospital in Bahrain and did not include other inpatients from private institutes. Hospital characteristics were not taken into consideration when assessing factors affecting LOS. Furthermore, data included inpatients admitted during a one-year period in acute psychiatric wards, which is a short period and did not include patients in long-stay wards; thus it is difficult to generalize the results of the study.

## Conclusions

In conclusion, patients who were diagnosed with psychotic disorders as well as bipolar affective disorders had longer hospital stays. The number of previous admissions, multiple medications, and history of side effects to medications are also other important factors affecting length of stay. Gender, age, and marital status were insignificant factors. Future studies should focus on the effect of better treatment options as well as occupational rehabilitation in ensuring better outcomes for inpatients as well as shorter stays in a psychiatric hospital.

## Appendices

Participant form

Demographic information:

Name:

CPR (Central Population Registry) number:

Age:

Gender:

-      Male

-      Female

Marital status:

- Single

- married

- Divorced

- Widowed

Religion:

Nationality:

-      Bahraini

-      Non-Bahraini

Employment:

-      Student

-      Employed

-      Unemployed

-      Retired

Social class (SC):

-      SC1

-      SC2

-      SC3

-      SC4

-      SC5

History during admission:

History of restraints during current admission: - Yes - No - Number of restraints

History of PRN (pro re nata) received: - How many times received - Type of PRN received

Presence of extrapyramidal side effects during admission:

Medications used during admission:

-      Initial medication and dose

-      Discharge medication

History of discharge:

Discharge diagnosis:

Length of stay:

Patient condition at discharge:

-      Improved

-      Discharge against medical advice

-      Away without leave

Other information:

History of suicide

History of homicide

Number of previous admissions:

Number of admissions this year

History of electroconvulsive therapy (ECT)

-      Yes

-      No

Comorbid medical and psychiatric conditions:

Smoking:

-      Yes

-      No

Alcohol:

-      Yes

-      No

Substance abuse:

-      Yes

-      No
